# 1152. Quantifying the health equity related public health impact of national immunisation programmes

**DOI:** 10.1093/ofid/ofad500.993

**Published:** 2023-11-27

**Authors:** Eliana Biundo, Mariia Dronova, Annie Chicoye, Richard Cookson, Nancy Devlin, T Mark Doherty, Antonio J Garcia-Ruiz, Louis P Garrison, Terry Nolan, Maarten Postma, David M Salisbury, Hiral Shah, Jurgen Wasem, Ekkehard Beck

**Affiliations:** GSK, Wavre, Brabant Wallon, Belgium; Putnam PHMR, Krakow, Dolnoslaskie, Poland; AC Health Consulting, Paris, Ile-de- France, France; Centre for Health Economics, York, England, United Kingdom; University of Melbourne, Melbourne, Australian Capital Territory, Australia; GSK, Wavre, Brabant Wallon, Belgium; University of Malaga, Malaga, La Rioja, Spain; The Comparative Health Outcomes, Policy, and Economics (CHOICE) Institute, Washington, Washington; The Peter Doherty Institute for Infection and Immunity, Murdoch Children’s Research Institute, Melbourne, Victoria, Australia; University of Groningen, Groningen, Groningen, Netherlands; Royal Institute of International Affairs, Chatham House, London, London, England, United Kingdom; GSK, Wavre, Brabant Wallon, Belgium; University of Duisburg-Essen, Essen, Hessen, Germany; GSK, Wavre, Brabant Wallon, Belgium

## Abstract

**Background:**

COVID-19 highlighted health inequities and the differential impact that vaccination can have on health, with respect to social advantage. While many national immunisation programmes (NIP) consider health equity as a key recommendation criterion, most lack robust frameworks and methods to quantify the impact of healthcare interventions on health equity. Equity strata can be defined by socioeconomic status, race/ethnicity, geographic location, etc…

**Methods:**

The inclusion of equity in guidelines for vaccination recommendation was assessed. Vaccination policy experts and health economists were consulted on how to best capture the equity-related public health impact (PHI) of NIP. The framework of distributional cost-effectiveness analysis (DCEA) was selected to be further explored. A stepwise approach was applied to account for equity in PHI analysis i.e., the impact on health outcome distribution by equity strata, by estimating the distributional impact (step 1) and HE impact (step 2) of vaccination. A practical application of the method was illustrated through a case study on the meningococcal B (MenB) disease vaccination program in England.

**Results:**

Impact of vaccination in health equity is formally captured in policy decision making in the US ACIP Evidence to Recommendation framework and the Canadian EEFA (Ethics, Equity Feasibility, Acceptability) Framework. In UK, France, Australia, Spain and Germany it is qualitatively included as part of the deliberative process, while it is not captured in the Netherlands. The DCEA framework was applied for health equity benefits of MenB infant vaccination in UK, with impact assessed across population subgroups categorised using a deprivation index. Nearly 80% of prevented cases were among the three most deprived groups. Additionally, MenB vaccination decreased inequity in the population, with positive net equity impact.

**Conclusion:**

PHI of vaccination on health equity can be quantified and usefully considered in decision making. The case study demonstrates how the proposed framework could be applied to fully incorporate impact on equity in cost-effectiveness analysis. The health equity impact of vaccination can be captured in health economic evaluation although there is a need to improve the evidence base and its implementation

**Disclosures:**

**Eliana Biundo, PhD**, GSK: employee|GSK: Stocks/Bonds **Mariia Dronova, PhD**, GSK: Grant/Research Support **Annie Chicoye, PhD**, GSK: Board Member|GSK: Grant/Research Support|GSK: Honoraria **Richard Cookson, PhD**, Genetech: Advisor/Consultant **Nancy Devlin, PhD**, GSK: Honoraria **T. Mark Doherty, PhD**, GSK: employee|GSK: Stocks/Bonds **Antonio J Garcia-Ruiz, PhD**, CHIESI: Advisor/Consultant|CHIESI: Grant/Research Support|CHIESI: Honoraria|Consumers and Users Organisations (CEACCU): Advisor/Consultant|Consumers and Users Organisations (CEACCU): Grant/Research Support|Consumers and Users Organisations (CEACCU): Honoraria|Foundation for Progress and Health (Andalusian Regional Government): Advisor/Consultant|Foundation for Progress and Health (Andalusian Regional Government): Grant/Research Support|Foundation for Progress and Health (Andalusian Regional Government): Honoraria|GSK: Honoraria|Ministry of Science and Innovation of Spain: Advisor/Consultant|Ministry of Science and Innovation of Spain: Grant/Research Support|Ministry of Science and Innovation of Spain: Honoraria|Official College of Physicians: Advisor/Consultant|Official College of Physicians: Grant/Research Support|Official College of Physicians: Honoraria|Royal Academy of Medicine and Surgery of Eastern Andalusia: Advisor/Consultant|Royal Academy of Medicine and Surgery of Eastern Andalusia: Grant/Research Support|Royal Academy of Medicine and Surgery of Eastern Andalusia: Honoraria|Sanofi Pasteur: Advisor/Consultant|Sanofi Pasteur: Grant/Research Support|Sanofi Pasteur: Honoraria|Sociedade Galega de Neuroloxía: Advisor/Consultant|Sociedade Galega de Neuroloxía: Grant/Research Support|Sociedade Galega de Neuroloxía: Honoraria|UCB: Advisor/Consultant|UCB: Grant/Research Support|UCB: Honoraria **Louis P Garrison, PhD**, GSK: Honoraria **Terry Nolan, MD, PhD**, Clover: Board Member|CSL Seqirus: Advisor/Consultant|CSL Seqirus: Grant/Research Support|Dynavax: Grant/Research Support|GSK: Advisor/Consultant|GSK: Board Member|GSK: Grant/Research Support|Iliad: Grant/Research Support|Moderna: Advisor/Consultant|Moderna: Grant/Research Support|MSD: Advisor/Consultant|MSD: Grant/Research Support|Novavax: Board Member|Pfizer: Advisor/Consultant|Sanofi: Advisor/Consultant|Sanofi: Grant/Research Support|SK Bio: Board Member **Maarten Postma, Dr.**, Consumers and Users Organisations (CEACCU): Advisor/Consultant|Consumers and Users Organisations (CEACCU): Honoraria|Foundation for Progress and Health (Andalusian Regional Government): Advisor/Consultant|Foundation for Progress and Health (Andalusian Regional Government): Honoraria|GSK: Honoraria|Royal Academy of Medicine and Surgery of Eastern Andalusia: Advisor/Consultant|Royal Academy of Medicine and Surgery of Eastern Andalusia: Honoraria **David M. Salisbury, CB FMedSci FRCP FRCPCH FFPH**, Clover Pharmaceuticals: Advisor/Consultant|GSK: Advisor/Consultant|Moderna: Advisor/Consultant|Novavax Inc: Honoraria|Sanofi: Advisor/Consultant **Hiral Shah, PhD**, GSK: employee|GSK: Stocks/Bonds **Jurgen Wasem, PhD**, AstraZeneca: Advisor/Consultant|AstraZeneca: Honoraria|Clover: Advisor/Consultant|Clover: Board Member|GSK: Board Member|GSK: Honoraria|Merck: Advisor/Consultant|Merck: Honoraria|Sanofi Pasteur: Advisor/Consultant|Sanofi Pasteur: Honoraria|Seqirus: Advisor/Consultant|Seqirus: Honoraria|Serum Institute of India: Advisor/Consultant|Serum Institute of India: Board Member|Zeria: Advisor/Consultant|Zeria: Board Member **Ekkehard Beck, PhD**, GSK: employee|GSK: Stocks/Bonds

